# FTIR Analysis of Renal Tissue for the Assessment of Hypertensive Organ Damage and proANP_31–67_ Treatment

**DOI:** 10.3390/ijms24065196

**Published:** 2023-03-08

**Authors:** Leonardo Pioppi, Niki Tombolesi, Reza Parvan, Gustavo Jose Justo da Silva, Raffaele Altara, Marco Paolantoni, Assunta Morresi, Paola Sassi, Alessandro Cataliotti

**Affiliations:** 1 Department of Chemistry, Biology and Biotechnology, University of Perugia, 06123 Perugia, Italy; 2 Institute for Experimental Medical Research, Oslo University Hospital and University of Oslo, 0450 Oslo, Norway

**Keywords:** FTIR imaging, kidney disease, vastiras, glomeruli, renal tubules

## Abstract

The kidneys are one of the main end organs targeted by hypertensive disease. Although the central role of the kidneys in the regulation of high blood pressure has been long recognized, the detailed mechanisms behind the pathophysiology of renal damage in hypertension remain a matter of investigation. Early renal biochemical alterations due to salt-induced hypertension in Dahl/salt-sensitive rats were monitored by Fourier-Transform Infrared (FTIR) micro-imaging. Furthermore, FTIR was used to investigate the effects of proANP_31–67_, a linear fragment of pro-atrial natriuretic peptide, on the renal tissue of hypertensive rats. Different hypertension-induced alterations were detected in the renal parenchyma and blood vessels by the combination of FTIR imaging and principal component analysis on specific spectral regions. Changes in amino acids and protein contents observed in renal blood vessels were independent of altered lipid, carbohydrate, and glycoprotein contents in the renal parenchyma. FTIR micro-imaging was found to be a reliable tool for monitoring the remarkable heterogeneity of kidney tissue and its hypertension-induced alterations. In addition, FTIR detected a significant reduction in these hypertension-induced alterations in the kidneys of proANP_31–67_-treated rats, further indicating the high sensitivity of this cutting-edge imaging modality and the beneficial effects of this novel medication on the kidneys.

## 1. Introduction

Chronic kidney disease (CKD) continues to be a major health concern and a leading cause of death, with high blood pressure being a primary cause of renal failure [1,2]. CKD is characterized by tubular atrophy, vasculopathy, glomerulosclerosis, increased renal interstitial fibrosis, and reduced capacity for renal regeneration [3,4,5]. Renal damage and hypertension are known independent predictors of morbidity and mortality [6]. To date, there are no effective treatments to prevent the worsening of renal impairment, as few medications have been proven to have protective effects on the renal parenchyma. It has been suggested that the lack of effective therapies is, at least in part, due to the late diagnosis of CKD and that earlier detection of renal impairment could increase the efficacy of an intervention [7]. The histopathological analysis of kidney biopsies is the gold standard for an accurate diagnosis of renal damage [8,9]. Most of the late parenchymal lesions can be identified by microscopy [10]. However, the study of early renal biochemical alterations induced by hypertension is still an area of investigation. Fourier-transform infrared (FTIR) microspectroscopy allows for fast and label-free biochemical imaging of many biological tissues [11], and micro-FTIR analyses of the kidneys have accurately detected early biochemical alterations related to cancerous tissue [12,13] and other nephropathies [14,15]. Thus, this technique can be used to detect small changes in biochemical composition. However, the application of FTIR for the assessment of hypertension-related kidney lesions has not been reported. We recently demonstrated that vibrational spectroscopic techniques are efficient diagnostic tools that are capable of detecting early chemical changes that precede the structural and functional cardiac alterations induced by prolonged hypertension [16].

In the current study, we applied FTIR imaging with hierarchical cluster analysis (HCA) and principal component analysis (PCA) to assess, at the molecular level, the early structural changes in the kidney that are induced by chronic hypertension. To achieve this aim, we used Dahl/salt-sensitive (DSS) rats and monitored the kidney damage at six weeks of salt-induced hypertension. In the present work, FTIR analysis was also applied to investigate the effect of proANP_31–67_, a linear fragment of pro-atrial natriuretic peptide [17]. This drug was recently developed to enhance renal function [18]; however, its effects on renal tissue have not been fully characterized. Here, we investigated the actions of proANP_31–67_ on renal biochemistry and the sensitivity of FTIR for detecting such effects in DSS hypertensive rats. 

## 2. Results

### 2.1. Renal Functional and Structural Assessment

**Figure 1:**
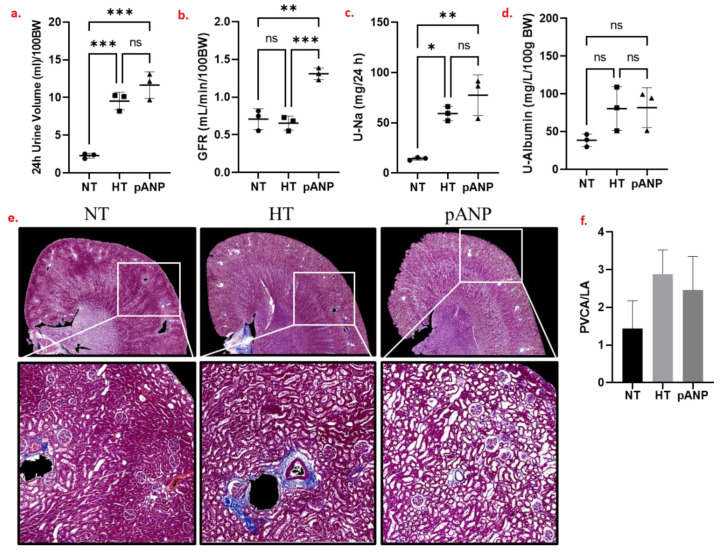
(**a**–**d**) Renal functional data obtained for NT (circles), HT (squares), and pANP (triangles) rats. Zero (n.s.) or low (*) to high (***) statistical significance for the differences between data are reported. (**e**) Masson’s trichrome staining of representative formalin-fixed paraffin-embedded (FFPE) kidney sections. (**f**) Perivascular collagen deposition. NT, normotensive; HT, hypertensive; pANP, proANP_31–67_-treated; BW, body weight; GFR, glomerular filtration rate; U-Na, urinary sodium; U-albumin, urinary albumin; PVCA/LA, perivascular collagen area/lumen area. The data are presented as mean ± SD.

Six weeks of a high-salt diet compared to a normal diet resulted in more pronounced diuresis, maintained glomerular filtration rate (GFR), higher sodium excretion, and tended to increase albuminuria (Figure 1a–d). The high-salt diet resulted in an increase in blood pressure, which was not reduced by proANP_31–67_. On histological analysis, the high-salt diet tended to increase fibrosis and perivascular collagen deposition (Figure 1e,f). 

### 2.2. Spectroscopic Characterization of Renal Tissue 

**Figure 2:**
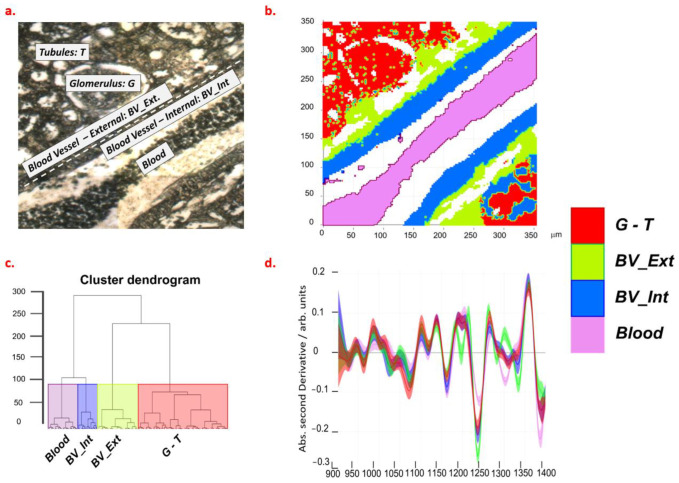
Optical (**a**) and FTIR (**b**) images of renal tissue from a normotensive rat. Results of HCA analysis are shown as a dendrogram (**c**) and spectra selected at cluster height > 100 (**d**). The violet, blue, green, and red colors of panel b, c, and d are, respectively, referred to as the 1–4 clusters of HCA. Legend: G, glomeruli; T, tubules; BV_Ext, external wall of blood vessel; BV_Int, internal wall of blood vessel.

Unstained histological slides were examined by conventional microscopy for the identification of kidney structure and compartments (Figure 2a). Hierarchical cluster analysis (HCA) of FTIR spectra revealed a clear segmentation of such compartments, depending on the spectral range used for the distance measurement: if a range between 900 cm^−1^ and 1400 cm^−1^ was used, clusters showed a different composition for blood, vessel walls (internal and external), and glomeruli/tubules (Figure 2b,c). The 900–1400 cm^−1^ range of IR absorption spectrum is characterized by the presence of signals characteristic of carbohydrates (1000–1150 cm^−1^), lipids (1150–1200 and 1330–1340 cm^−1^), proteins (1240–1300 cm^−1^), and free amino acids (1396 cm^−1^) [11,19]. 

To identify the mean composition of the different areas, we visually inspected the second derivative spectra that were obtained from classes segmented by HCA (Figure 1d).

These spectral profiles showed that blood is characterized by a high content of free amino acids and lipids; internal vessel walls by a high content of lipids and proteins; external walls by the highest content of proteins; glomeruli and tubules by the highest content of glycogen. Based on optical images and supported by the results of HCA, we selected spectra from regions identified as glomeruli, tubules, and vessel walls in the kidneys of NT, HT, and proANP_31–67_-treated rats. For the analysis of vessels, only the results from NT and HT tissues were compared as the patency of the proANP_31–67_-treated vessels did not allow for unambiguous interpretation. 

**Figure 3:**
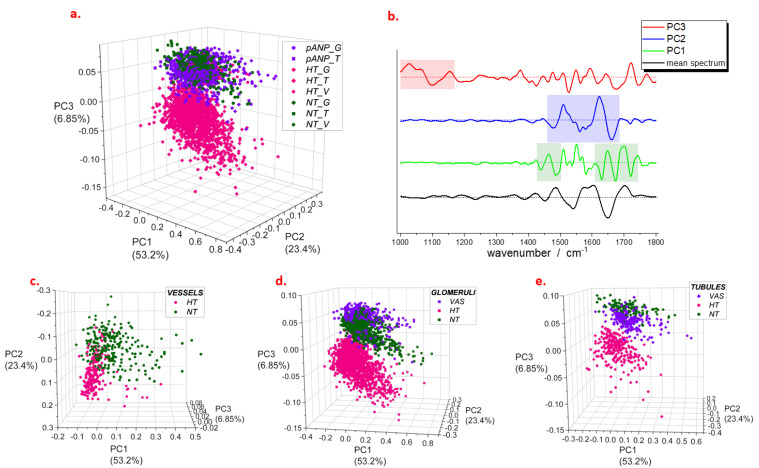
PCA scores (**a**) and loadings (**b**) from analysis of spectra selected on different structures of renal parenchyma. In panel (**b**), shadowed areas demonstrate the most intense contributions to the loadings; mean spectrum refers to the entire set of data; dashed lines indicate the zero intensity for each curve. Detail of scores obtained for vessels (**c**), glomeruli (**d**), and tubules (**e**). Legend: NT, normotensive; HT, hypertensive; pANP, proANP_31–67_-treated.

For a range between 1000 cm^−1^ and 1800 cm^−1^, principal component analysis (PCA) showed that the first three principal components (i.e., PC1, PC2, and PC3) explain more than 80% of the total variance (Figure 3a). 

For each of these, the most intense contributions were highlighted in the respective loading plots (Figure 3b). Regarding PC1, the most relevant regions are 1430–1480 cm^−1^ and 1620–1740 cm^−1^, which are assigned to both lipids (signals at 1430–1480 cm^−1^ and 1700–1740 cm^−1^) and proteins (Amide I band at 1620–1700 cm^−1^). The loadings of PC2 showed intense features in a region between 1450 cm^−1^ and 1700 cm^−1^ with a larger contribution from the protein signals (Amide II and Amide I bands at 1485–1700 cm^−1^); on the other hand, loadings of PC3 showed several distinct spectral features in a range between 1000 cm^−1^ and 1150 cm^−1^, which may be attributed to carbohydrates and glycoproteins. We found that a combination of PC1 and PC2 describes the major differences seen between the blood vessel spectra of NT and HT samples (Figure 3c). In particular, HT samples showed a shift in the positive direction of the PC2 score axis, suggesting that important changes in the protein content characterize vessel composition in HT compared to NT. Both PC2 and PC3 differentiate the positive scores of NT and proANP_31–67_-treated samples from the negative ones of HT in the spectral profiles of tubules and glomeruli (Figure 3d,e). The same marker bands were observed to differentiate the mean spectral profiles of HT from those of NH and pANP (Figure S1). The PCA of spectra from single anatomic groups also showed similar results (Figure S2), confirming the common features of glomeruli and tubules. Thus, for the latter, we selected a range between 1000 cm^−1^ and 1200 cm^−1^ to perform the analysis (Figure S3a) and observed that more than 60% of the total variance is explained by a component (PC1) with an intense feature at 1030 cm^−1^ (Figure S3b) whose scores are negative for NT and proANP_31–67_-treated, and positive for HT (both tubules and glomeruli) data. 

**Figure 4:**
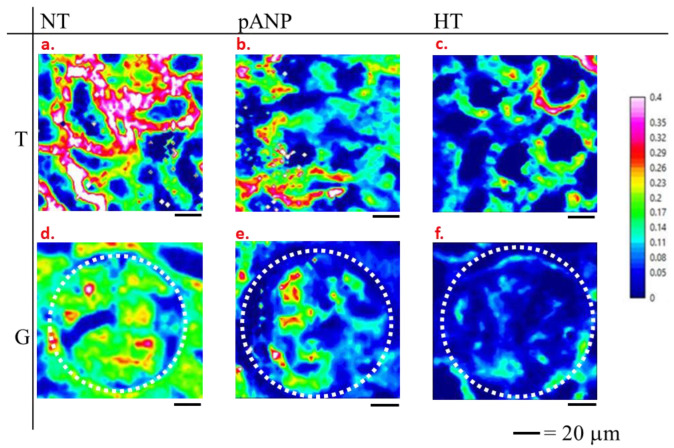
Examples of FTIR images of L/CG ratio for tubular (T) (**a**–**c**) and glomerular (G) (**d**–**f**) structures from normotensive (NT) (**a**,**d**), proANP_31–67_-treated (pANP) (**b**,**e**), and hypertensive rats (HT) (**c**,**f**). Glomerular units of panels d, e, f are delimited by the white dotted line.

Since the spectral markers of HT are located on specific bands of the FTIR spectrum (protein bands for blood vessels and glycoprotein/carbohydrate bands for glomeruli and tubules), we performed a univariate analysis of FTIR spectra by selecting specific marker bands to monitor the lipid (2840–2860 cm^−1^), protein (1485–1700 cm^−1^), glycoprotein/carbohydrate (1000–1128 cm^−1^), and free amino acid (1358–1416 cm^−1^) contents. In Figure 4, an example of FTIR images representing the ratio of integrated intensities of lipids to glycoprotein–carbohydrate (L/CG) bands is shown. The upper panels of Figure 4 shows the L/CG intensity ratio in tubules from NT, proANP_31–67_-treated, HT, and kidneys; the lower panels show the glomeruli of the same samples. All the panels were normalized to the same range of intensity ratio (scale on the right), which allows for a comparison of the composition of the different tissues with respect to the L/CG content. Both tubules and glomeruli of HT samples were associated with a reduced L/CG ratio compared to NT samples, whereas proANP_31–67_-treated samples showed values that were intermediate between NT and HT samples. 

**Figure 5:**
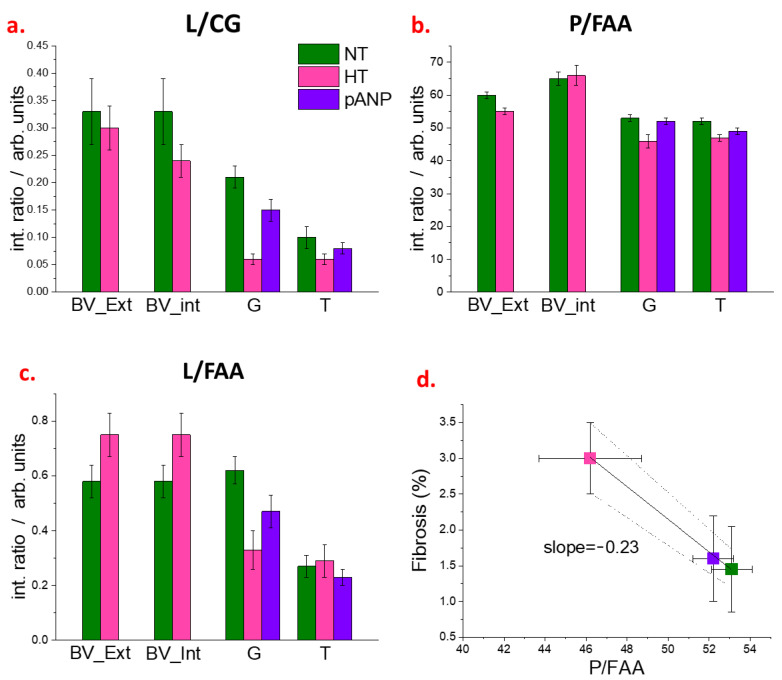
(**a**–**c**) Ratiometric analysis from FTIR spectra of NT, HT, and proANP_31–67_ samples. Integrated intensities of the lipid (L, 2840–2860 cm^−1^), protein (P, 1485–1700 cm^−1^), carbohydrate/glycoprotein (C/G, 1000–1128 cm^−1^), and free amino acid (FAA, 1358–1416 cm^−1^) marker bands are used to estimate L/CG, L/FFA, and P/FAA ratios for the external (BV_Ext) and internal (BV_Int) regions of blood vessels, for glomeruli (G) and tubules (T). (**d**), Linear regression for the renal fibrosis as assessed by Masson’s trichrome staining versus glomeruli P/FAA. The dotted lines indicate the 95% confidence range of the best-fit line. Legend: NT, normotensive; HT, hypertensive; pANP, proANP_31–67_-treated.

The intensity ratios (L/CG, P/FAA, L/FAA) between the different marker bands for internal and external regions of blood vessels, glomeruli, and tubules are displayed in Figure 5, as these structures have a different composition according to HCA and/or PCA. In HT kidneys, the L/CG was reduced in both the external and internal regions of blood vessels (BV_Ext and BV_Int, respectively) when compared to NT kidneys (*p* < 0.01). The P/FAA was reduced in the BV_Ext (*p* < 0.01) and tended to increase in the BV_Int of HT kidneys compared to NT (*p* < 0.05). The L/FAA in HT kidneys was markedly increased, both in the BV_Ext and BV_Int, compared to NT kidneys (*p* < 0.01). In the glomeruli, L/CG, P/FAA, and L/FAA were lower in HT and were restored with proANP_31–67_ compared to NT (*p* < 0.01). In the tubules, both L/CG and P/FAA tended to be lower in HT compared to NT and restored with proANP_31–67_ (*p* < 0.01), while L/FAA increased in HT compared to NT and was reduced with proANP_31–67_ (*p* < 0.05). A negative correlation was observed between the glomerular P/FAA ratio and the extent of kidney fibrosis (Figure 5d and Table S1) and no correlation was observed between the other renal parameters (i.e., GFR, 24-h urine excretion, urinary-Na excretion, and urinary-albumin excretion) with any of the intensity ratios (Table S1).

## 3. Discussion

In the current study, we investigated kidney structural changes in a rat model of hypertension using FTIR imaging. Using HCA, FTIR was able to distinguish the spectral features of primary renal structures, such as blood vessels, tubules, and glomeruli. The capability to distinguish these histological structures based on their spectral markers allows for structure-specific analysis. Although renal functional parameters (i.e., GFR, 24-h urine excretion, and urinary-Na excretion) remained within the normal range after six weeks of sustained hypertension, the renal parenchyma tended toward remodeling, and FTIR analysis revealed biochemical changes induced by hypertension on blood vessels, as well as changes in glomeruli and tubules. Specifically, changes in amino acids and protein content were observed in blood vessels, changes in lipid and carbohydrate/glycoprotein concentrations were detected in glomeruli, and changes in carbohydrate/glycoprotein content were observed in tubules. These changes, although associated with renal structural alterations, were not associated with the changes in renal function as indicated by GFR. Indeed, GFR was similar in the untreated HT and NT rats; thus, these alterations may constitute an early FTIR marker profile of hypertension-induced organ damage. Of note, treatment with proANP_31–67_ tended to improve renal structure compared to HT-untreated rats (Figure 1e,f). Importantly, FTIR detected these changes associated with proANP_31–67_-treatment and could differentiate between treated versus untreated HT kidneys (Figure 2, Figure 3, Figure 4 and Figure 5), even if the treatment did not reduce blood pressure. 

Fibrosis and vessel wall thickening are common histological alterations encountered as a response to hypertension [20]. In the PCA of our spectroscopic data, more protein and free amino acids were detected in HT vessel walls. These results may be explained by the progression of fibrosis. The FAA signal at 1396 cm^−1^ is characteristic of free amino acids as well as terminal COO- groups of protein molecules [21,22]. An increase in intensity for the FAA and P bands is likely attributed to increased collagen concentration; in particular, the FAA signal could relate to the short-chain collagen fraction. Taken together, the decreased P/FAA value in HT samples as compared to NT samples (Figure 5), and the negative correlation between P/FAA and the level of fibrosis (Figure 5d), suggest that the short-chain species increase is greater than the increase in long-chain collagen. In a recent study, a significant amount of perivascular collagen deposition (perivascular fibrosis) was observed in the kidney cortex of HT compared to NT rats [18]. Our data confirmed these findings and demonstrated that the molecular structures of the deposits are rich in amino acids/short-chain proteins. One possible explanation for this finding is the accumulation of non-fibrillar short-chain type VIII collagen in the outer vessel walls, as previously reported in diabetic nephropathy [23]. 

Second-harmonic-generation microscopy of human and mouse kidneys has revealed that nephropathy-related fibrosis and glomerulosclerosis correspond to an accumulation of different extracellular proteins [24]. Our results showed that, with hypertension, different biochemical changes occurred in vessels as compared to glomeruli and tubules. Recent findings in hypertensive DSS rats revealed that metabolic stress primarily affects glomeruli, whereas tubules showed changes related to altered amino acid handling [25]. The strong decrease in L/CG and L/FAA ratios that we observed was more prominent in glomeruli than in tubules (see Figure 5). This finding is consistent with prior reports of a reduction in glomerular lipid membranes in the setting of hypertension, perhaps due to oxidation processes [26]. 

The decrease in the L/CG ratio may also be a consequence of altered metabolism, as has been previously shown by Wang et al. [27] in a similar rat model. Following the CG marker band, univariate analysis and PCA indicated that the kidney parenchyma of HT rats has a higher carbohydrate and/or glycoprotein content compared to NT rats. This alteration affected both glomeruli and tubules and was substantially prevented by the administration of proANP_31–67_ (Figure 5a). Using isolated glomeruli from DSS hypertensive rats, Domondon et al. observed a decline in mitochondrial respiratory function, together with increased oxidative stress and reduced antioxidant capacity [26]. This impairment in mitochondrial function could explain our observation that carbohydrates accumulate in these structures. The increase in carbohydrate and glycoprotein content might also be explained by chronic glomerular ischemia and subsequent tubular atrophy induced by early hypertensive kidney damage [28]. 

Our results indicated that hypertension-related changes in carbohydrate and glycoprotein contents of the renal parenchyma are readily detectable by FTIR analysis. ProANP_31–67_ treatment resulted in normalization of the carbohydrate, protein, and amino acid content in all analyzed renal compartments. These effects were associated with a reduction in fibrosis, as assessed by conventional histological analysis. These favorable effects on renal chemical components and the reduced remodeling both resulted in enhanced GFR. Despite the clear renal protective effects of proANP_31–67_, it is possible that proANP_31–67_ had other direct effects on the renal vasculature, as we observed altered patency of the vessels in the kidneys of the treated animals. The pathophysiological consequences of such altered vascular patency were not investigated as they were beyond the scope of the current investigation. 

## 4. Materials and Methods

### 4.1. Experimental Group and Study Protocol

The experimental protocol was approved by the Committee for Animal Research of the Norwegian Food Safety Authority (Mattilsynet, protocol number 12582). DSS rats were purchased from Charles River Laboratories (Wilmington, MA, USA) and housed in a room with a 12:12 h light:dark cycle at a temperature of 21 °C and a humidity level of 55%. Consistent with the American Veterinary Medical Association (AVMA) Guidelines for the Euthanasia of Animals (2020), animals were sacrificed via deep anesthesia (5% isoflurane), exsanguination, and organ excision. 

Nine DSS male rats (~150 g initially) were used; three rats per group were recognized as the minimum number of animals needed to achieve reproducibility in terms of results, given the established consistency of the model and the high sensitivity of FTIR to monitor the chemical composition of cells and tissues [13,15,19]. The DSS rat develops high blood pressure within two days and cardiorenal symptoms within four weeks when chronically fed a high-salt diet [18]. All rats were given a normal (0.3% NaCl) salt diet (Special Diets Services, United Kingdom) until seven weeks of age. At that stage, three rats were kept normotensive (NT), while six were fed a high-salt (4% NaCl) diet (Envigo, TD.92034; Madison, WI, USA) for six weeks to induce hypertension. Starting four weeks before sacrifice (two weeks after initiation of the high-sodium diet), three hypertensive (HT) rats were chronically treated with subcutaneous (s.c.) infusion of proANP_31–67_ (50 ng/kg per day) via an Alzet osmotic mini-pump and three served as HT controls receiving vehicle. Parameters of renal function were assessed as we previously described [18]. 

### 4.2. Histochemistry

Kidneys were excised, rinsed in PBS and blotted on gauze, and fixed in 10% formalin for 24 h. The right kidney was embedded in paraffin and cut into 4 µm sections, which were stained with Masson’s trichrome (Polysciences, Inc., Warrington, PA, USA) to assess collagen deposition. Stained sections were scanned (20× magnification) with AxioScan Z1 (Carl Zeiss, Jena, Germany) to obtain whole cross-sections for collagen quantification. Perivascular fibrosis was defined as the area of collagen surrounding the vessel wall indexed to the vessel lumen area (PVCA/LA), averaged over all quantifiable images of vessels taken from the renal cortex per section (mean, 4.73 ± 0.88 quantifiable images of arteries). Only rounded (not collapsed) vessels were used for quantification. Quantification was performed using ZEN2 blue edition (Carl Zeiss). All histological quantifications were performed independently by an experienced researcher who was blinded to group identity.

### 4.3. FTIR Measurements

Deparaffined tissue sections were used. FTIR spectra were collected in transmission mode by using a Bruker Tensor 27 spectrometer and a Hyperion 3000 microscope equipped with a 15× Cassegrain objective and a 64 × 64 pixel focal-plane-array detector. The spectral range between 900 cm^−1^ and 3800 cm^−1^ was recorded at 6 cm^−1^ resolution. Spectral images of 180 µm × 180 µm with a pixel resolution of 2.8 µm were collected by averaging 256 measurements. At least three different areas from both medullary and cortical regions of each sample were analyzed, with particular reference to tubules and renal corpuscles. To reduce the influence of the baseline, which is remarkably variable due to the irregularity of the tissue, FTIR images were processed in second derivative spectroscopy; before HCA and PCA, a vector normalization in the 900–1800 cm^−1^ range was executed. IR spectrum of proANP_31–67_ was also measured and compared to tissue spectra; due to the low peptide concentration, we did not find any clear indication of proANP absorptions in the spectra of treated samples (Figure S4).

### 4.4. Statistical Analysis

The spectral dataset was analyzed using a proprietary R-package specifically developed for the hyperspectral analysis of big datasets, which includes tools for HCA and PCA [29]. Both procedures were initially tested on the entire spectral range (900–3800 cm^−1^) and then restricted to subranges to enhance the sensitivity of each technique after exploring several options. HCA was used on FTIR images of control samples to identify different renal structures, selecting the 900–1400 cm^−1^ spectral range for the distance measurement. The dataset of second derivative spectra obtained at each pixel of FPA exposure was processed to the computation of distance matrix by the Euclidean method and clusterization using Ward’s method. PCA was applied to include the different types of samples (NT, HT, and proANP_31–67_) after the selection of the spectra from different renal structures, such as internal and external regions of vessels, glomeruli, and tubules. These selections were guided by optical images and submitted to quality tests. The spectra arising from the empty spaces in the samples were rejected by setting a minimum threshold equal to 1/10 of the maximum integral intensity of the Amide I absorption band at 1620–1700 cm^−1^. Spectra with insufficient signal-to-noise ratio were also treated according to a quality test based on the intensity of the Amide I peak and the noise in a region between 1800 and 1900 cm⁻¹. We rejected spectra having a ratio lower than 80. The 1000–1800 cm^−1^ and 1000–1200 cm^−1^ spectral ranges were used for PCA of the entire dataset; the 1000–1800 cm^−1^ range was also used to analyze the spectra of specific tissue structures, i.e., vessels, glomeruli, and tubules. In both cases, a centered, unscaled principal component analysis was executed to obtain loadings and scores. With respect to the spectra treated by PCA, a univariate analysis was also performed by an estimate of integral intensities in the 2840–2860, 1485–1700, 1000–1128, and 1358–1416 cm^−1^ spectral regions. Integration of peak areas was performed by Opus 8.1 software from Bruker Optics; mean values and standard deviations of integrated area, correlation, and statistical significance by estimation of the p value were obtained by OriginPro 2021b software from OriginLab Corporation, Northampton, MA, USA. The sequence of data manipulation is described in Figure S5. 

## 5. Conclusions

In the present study, we report that FTIR spectroscopy provided molecular images of renal tissue that demonstrate alterations secondary to early-stage hypertension. Renal structures, such as blood vessels, glomeruli, and tubules, were chemically distinguished without any staining by assessing FTIR spectral features using HCA. We identified different spectral markers of hypertension in these structures. Univariate and multivariate analyses confirmed the same result, indicating that FTIR spectroscopy is an efficient and selective diagnostic tool for early renal tissue alterations. The major biochemical alterations after six weeks of sustained hypertension were detected in the renal parenchyma. Specifically, HT tubules and glomeruli showed an increase in carbohydrate and glycoprotein content as compared to NT. Of note, FTIR was able to detect the effect of chronic therapy with proANP_31–67_, which showed potential beneficial effects on the kidney function and structure. Remarkably, we observed a moderate biochemical change in carbohydrate and glycoprotein content in proANP_31–67_-treated rats, confirming its protective effect and opening new avenues of investigation into its mechanisms of action. Our study supports the potential utility of FTIR as an innovative method for the detection of early chemical changes in the renal parenchyma. These changes are otherwise underdiagnosed or missed as they precede changes in renal function such as a reduction in GFR. FTIR assessment of these chemical modifications of renal tissue could be useful to monitor the evolution of renal disease as well as the effects of therapeutic interventions.

## Data Availability

The data presented in this study are available on request from the corresponding author.

## Supplementary Materials

The following supporting information can be downloaded at: https://www.mdpi.com/article/10.3390/ijms24065196/s1.
