# Supplementary material for: FTIR Analysis of Renal Tissue for the Assessment of Hypertensive Organ Damage and proANP31–67 Treatment
Source: Int J Mol Sci. 2023 Mar 8;24(6):5196. doi: 10.3390/ijms24065196 (PMC10049716; doi:10.3390/ijms24065196)
Supplement: Supplementary file 1 [file ijms-24-05196-s001.zip › ijms-2215194-supplementary.pdf]

Supplementary Material

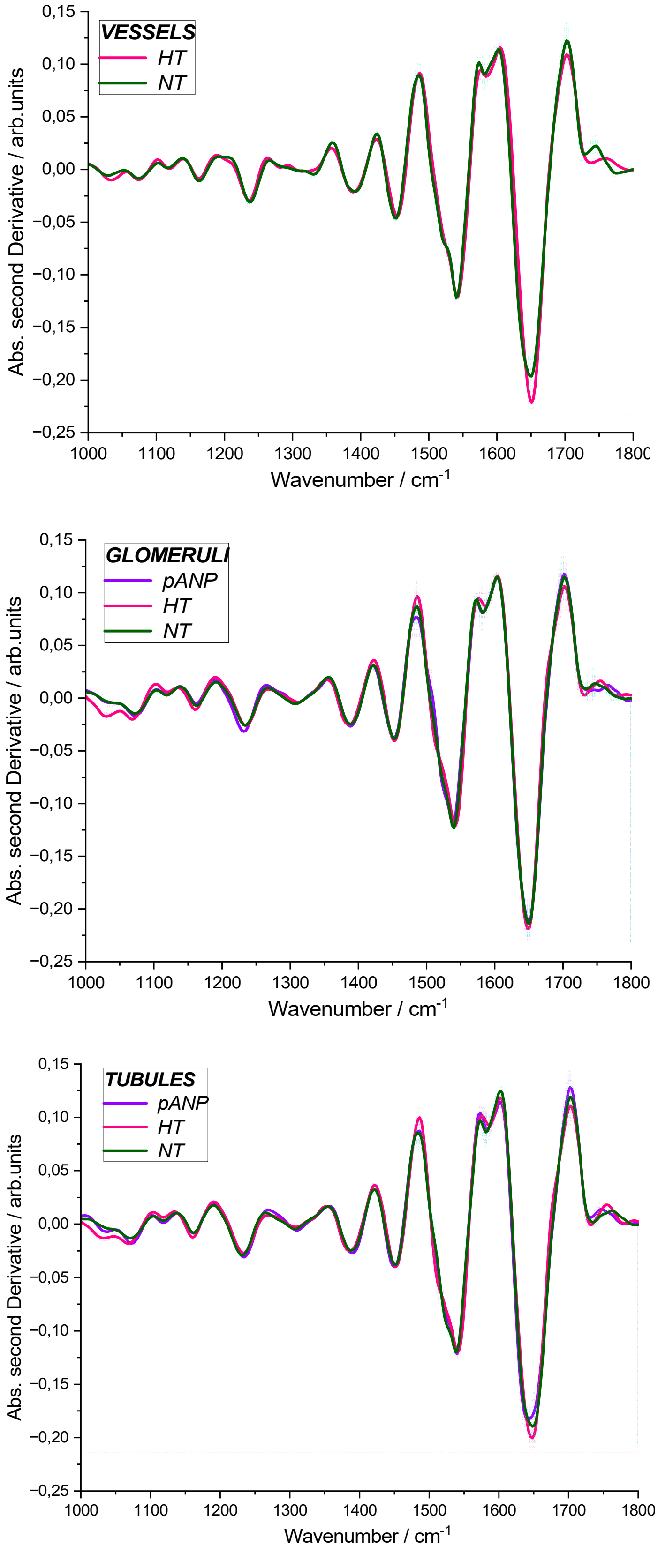

Figure S1. Mean second derivative profiles (with standard deviations reproduced as shadowed areas) of different renal structures, i.e. vessels, glomeruli, and tubules. The comparison of data shows that spectra obtained for tissues of NT and pANP samples are very similar in the whole 1000-1800 cm^{-1} range. Differently, HT profiles show significative differences in the intensity of

Amide I band of vessels (1620-1680 cm^{-1}), and in the carbohydrates/glycoprotein bands of glomeruli and tubules (1000-1180 cm^{-1}).

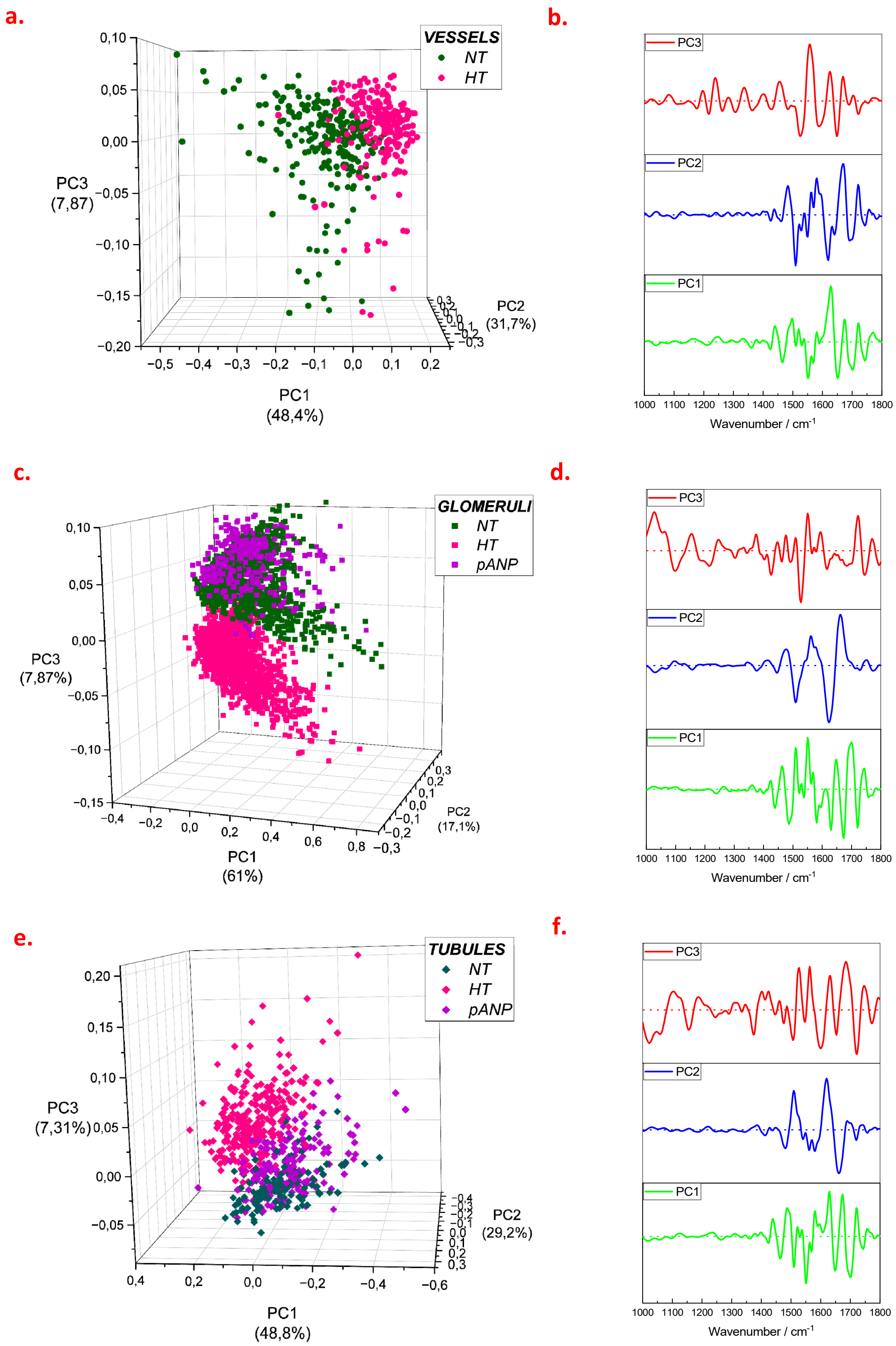

Figure S2. PCA scores (a,c,e) and loadings (b,d,f) from separate analysis of 1000-1800 cm^{-1} spectral range of vessels (a,b), glomeruli (c,d), and tubules (e,f). Different loadings were obtained for the different structures. The most significative contributions to PC1 and PC2 second derivative profiles are very similar to those obtained with the PCA of the entire dataset (see Fig.3): the only difference is the opposite sign of loadings and scores of PC2 in the case of tubules. A similar conclusion holds for PC3 of glomeruli and tubules. The third component obtained for vessel spectra does not display

a high intensity at 1000-1150 cm-1, indicating that carbohydrate/glycoprotein content does not influence NT and HT vessel differentiation as observed with the previous PCA.

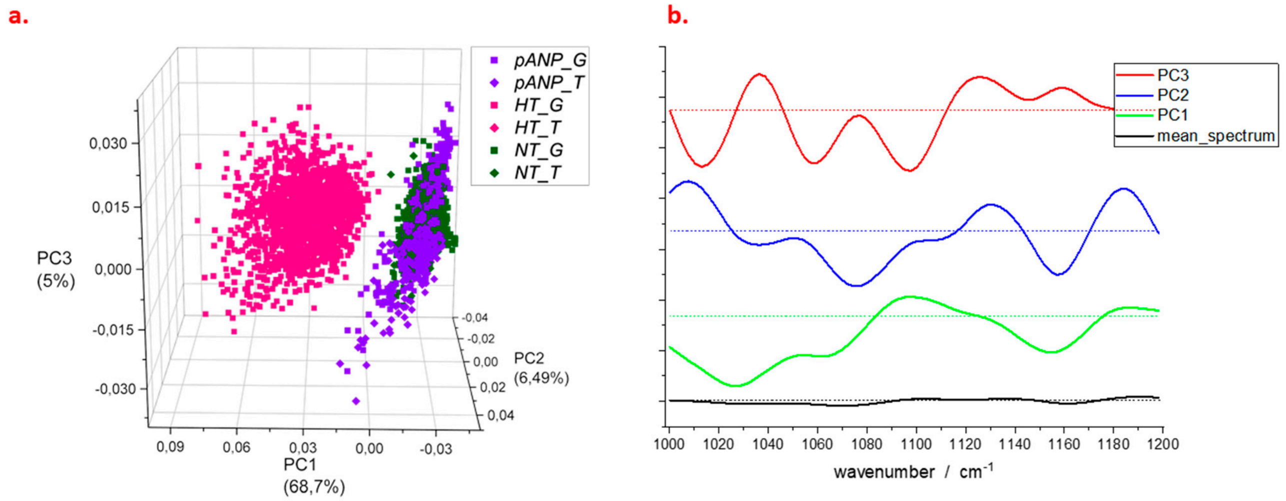

Figure S3. PCA scores (a) and loadings (b) from analysis of 1000-1200 cm-1 spectral range of entire dataset.
Dashed lines indicate the zero intensity in each PC plot.

Table S1. Significant correlations – p values

	Glomeruli L/CG	Glomeruli P/FAA	Glomeruli L/FAA	Tubules L/CG	Tubules P/FAA	Tubules L/FAA
GFR (mL/min)	ns	ns	ns	ns	ns	ns
U-Albumin (mg/L/100 g BW)	ns	ns	ns	ns	ns	ns
Kidney fibrosis	ns	0.0147*	ns	ns	ns	ns

GFR, glomerular filtration rate; U-Albumin, urine albumin

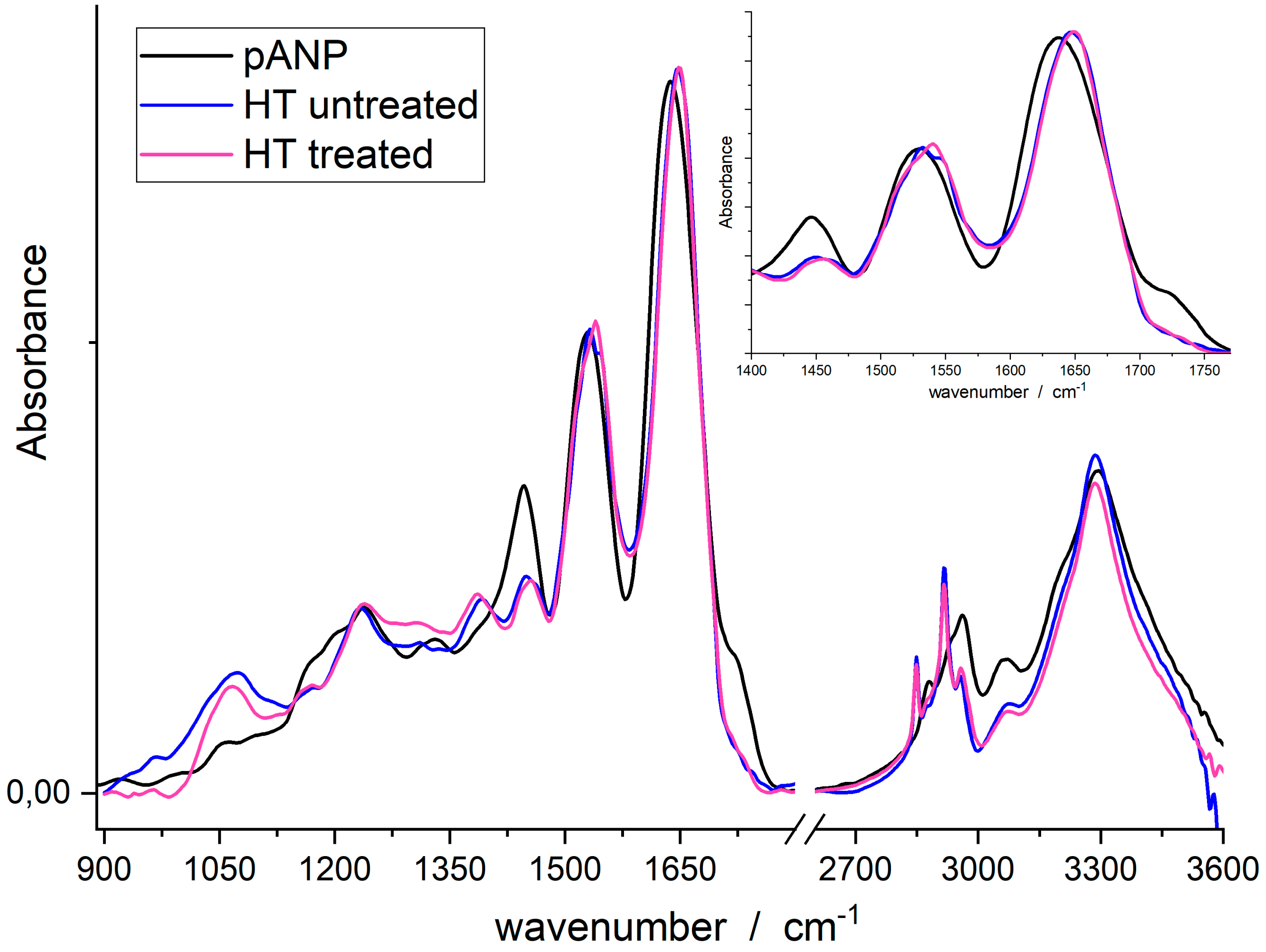

Figure S4. FTIR spectra obtained for glomeruli of proANP-treated and untreated rats are shown and compared to the one of proANP: there is no clear signature indicating the presence of the drug in the spectrum of pANP samples. Similar results were also obtained for tubules.

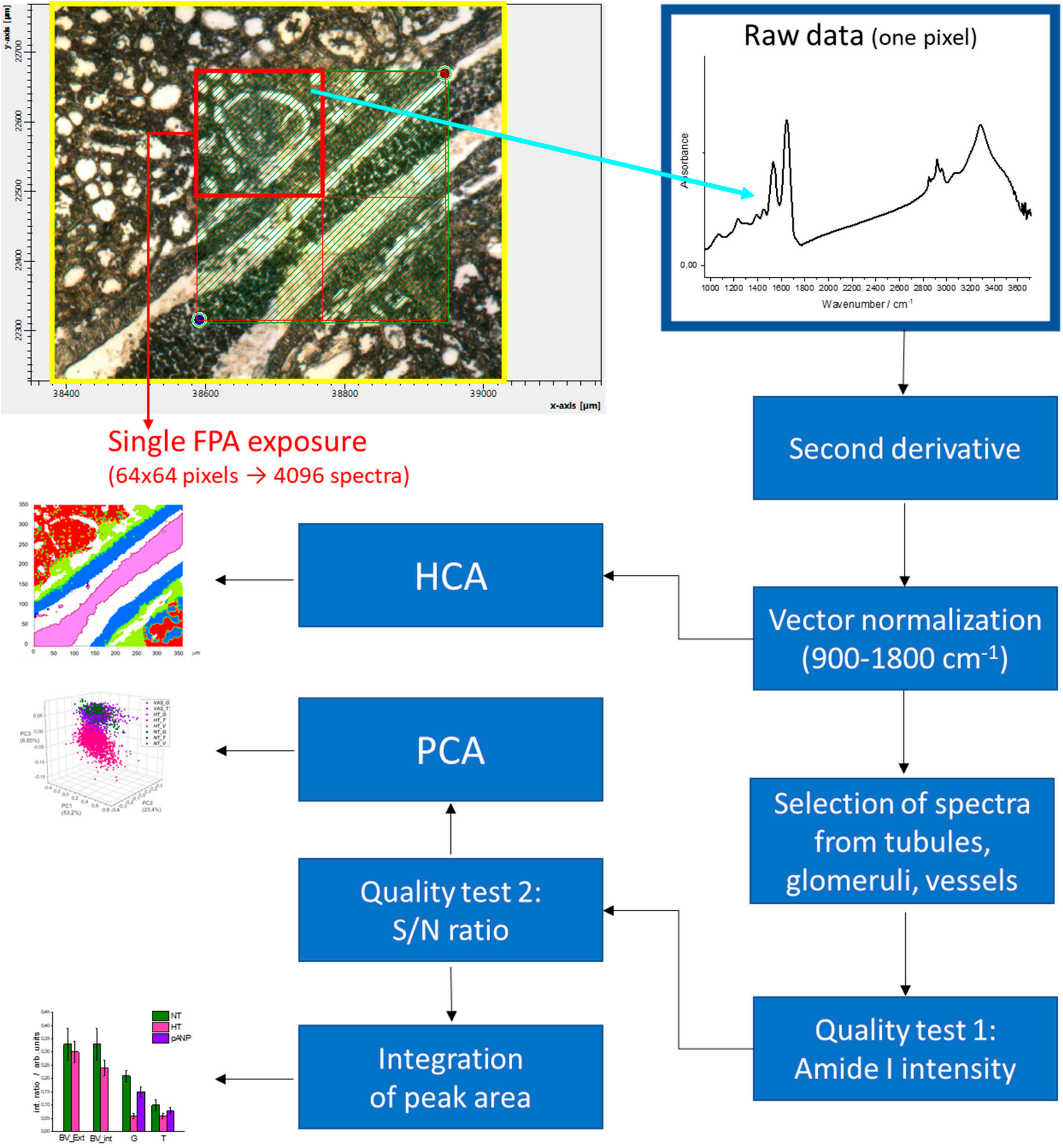

Figure S5. Flow chart of spectra manipulations
